# Gene Expression Profiling Reveals New Pathways and Genes Associated with Visna/Maedi Viral Disease

**DOI:** 10.3390/ani11061785

**Published:** 2021-06-15

**Authors:** Naiara Abendaño, Aitor Esparza-Baquer, Irantzu Bernales, Ramsés Reina, Damián de Andrés, Begoña M. Jugo

**Affiliations:** 1 Genetics, Physical Anthropology and Animal Physiology Department, Faculty of Science and Technology, University of the Basque Country (UPV/EHU), 48940 Leioa, Spain; naiara.carbajo@gmail.com (N.A.); aitor.esparzab@gmail.com (A.E.-B.); 2 Gene Expression Unit, Genomics Facility of General Research Services (SGIker), Faculty of Science and Technology, University of the Basque Country (UPV/EHU), 48940 Leioa, Spain; irantzu.bernales@ehu.eus; 3 Instituto de Agrobiotecnología (CSIC-Gobierno de Navarra), 31192 Mutilva Baja, Spain; ramses.reina@csic.es (R.R.); damian.deandres@csic.es (D.d.A.)

**Keywords:** Visna/Maedi, sheep, infection, pathogenesis, microarray, expression profiling

## Abstract

**Simple Summary:**

Visna/Maedi is a disease caused by a small ruminant lentivirus (SRLV), with different symptoms in adult sheep such as pneumonia, arthritis, encephalitis and mastitis. SRLV infection in sheep is widespread across the world, with Europe showing the highest individual prevalence. There is currently no effective treatment for SRLV infections and, due to their constant changes, effective vaccine development has been and is still challenging. The dynamics of the sheep immune response to these virus infections is unclear, and changes in gene expression can help to explain the processes occurring in infected sheep. In this study, a gene expression microarray was used to identify the differentially expressed genes in infected and diseased sheep by comparing animals with different serologic statuses and with the presence of VM-characteristic clinical lesions in the lungs. The expression profile analysis revealed many interesting genes that may be associated with the viral infection process (such as OXT and a number of genes implicated in the Toll Like Receptors signaling network and complement pathway). This work improves our understanding of the sheep immune response against SRLVs.

**Abstract:**

Visna/Maedi virus (VMV) is a lentivirus that infects the cells of the monocyte/macrophage lineage in sheep, goats and wild ruminants. Infection with VMV causes a multisystemic inflammatory disorder, which includes pneumonia, encephalitis, mastitis or arthritis. The immune response to VMV infection is complex, and the infection and pathogenesis of this virus are not totally characterized yet. In this work, a gene expression microarray was used to identify the differentially expressed genes in VMV infection and disease development by comparing sheep with different serologic status and with presence of VM-characteristic clinical lesions. The expression profile analysis has revealed many interesting genes that may be associated with the viral infection process. Among them, the OXT gene appeared significantly up-regulated, so the oxytocin-secreting system could play an essential role in VM disease. Moreover, some of the most significantly enriched functions in up-regulated genes appeared the complement pathway, which (in combination with the Toll-like receptor signaling network) could compose a mechanism in the VMV pathogenesis. Identifying the host genetic factors associated with VMV infection can be applied to develop strategies for preventing infection and develop effective vaccines that lead to therapeutic treatments.

## 1. Introduction

The sheep disease Visna/Maedi (VM) is one of the most important chronic health problems with respect to sheep production in the world. It is caused by the VM lentivirus, which, together with the caprine arthritis–encephalitis virus (CAEV), forms the SRLV group [1]. Animals become infected when young via the oral and respiratory routes, and afterwards the infection progresses slowly and clinical manifestations appear mainly in adults. SRLV infection is widespread across the world, having the highest individual prevalence (40.9%) in Europe and a prevalence higher than 15% in Africa, Asia and North America [2]. The infection usually causes multi-organ failure, leading to serious diseases such as pneumonia, mastitis, arthritis, wasting and various neurological disorders [3]. After Visna/Maedi Virus (VMV) infection, the animals can remain as asymptomatic carriers during their life, and only a few seropositive animals will develop clinical symptoms. VM is a disease that continues for a period of months or years, and always involves the death of the host, with the resultant economic and welfare effects [4].

The vast majority of works analyzing the gene expression profile in VM infection and disease have been performed in a gene-by-gene approach. Woodall et al. (1997) [5] found interleukin genes IL1, IL4, IL10 and interferon-gamma (IFNγ) to be up-regulated in the lungs of animals with clinical signs of VM. Members of the chemokine family (such as IL8 and chemokine receptor 5 (CCR5)) have been found to be involved in VMV infection and disease progression [6,7]. In addition, TNF and IL2 have been found to be down-regulated in the lungs of animals with clinical disease [8].

As far as the innate immunity is concerned, the expression of Toll Like Receptors (TLRs) and restriction factors such as APOBEC and TRIM5 have also been associated with VM. Larruskain et al. [8] found TLR7 and TLR8 up-regulated in the lungs of animals with lesions compared to control animals. Crespo et al. [9] analyzed the relative expression and sequences of TRIM5α, APOBEC3 (Z1, Z2, Z3 and Z2-Z3) and BST-2 and found a global antiviral status in low pro-viral carriers that may confer protection against viral shedding and disease onset. Only two works have searched for gene expression differences in seropositive versus seronegative animals. Thompson et al. [10] designed an experimental infection in goat synovial membrane cells and used RNAseq to detect a number of factors associated with MV infection (such as chemokine ligands CCL2, CCL5 and CCL20 and cytokines such as IL6, IL8 and IL16), all of them significantly modulated. Some known lentiviral restriction factors were also affected, tetherin (BST-2) among them. In a very recent work, Plawinska–Czarnak et al. [11] designed a custom transcriptomic array with more than 50,000 unique transcripts to detect the gene expression profile in goat PBMCs, but, strangely, only three genes were differentially upregulated: GIMAP2, SSC5D and SETX, and one downregulated: GPR37. These results suggest an active inflammatory mechanism in SRLV-positive goats.

Gene expression microarrays usually allow for the simultaneous measurement of the expression levels of thousands of genes and the identification of up- or down-regulated genes, and they have been used in sheep genomics to characterize gene expression in viral diseases [12] but mainly in bacterial diseases [13,14,15,16,17].

In the present work, we have used a commercial Agilent Sheep Gene Expression microarrays, 8 × 15 K (Agilent Technologies, Santa Clara, CA, USA), with 15,208 sheep features represented (including many immune-relevant genes) to find out the gene expression profiling associated with both VMV infection and disease lesions in sheep lungs.

## 2. Materials and Methods

### 2.1. Animals

A total of 33 Rasa Aragonesa adult (>2 years) ewes were included in this study, in different stages of a natural infection of VMV. Animals were classified according to their VMV infection status (seronegative or seropositive) using an Enzyme-Linked ImmunoSorbent Assay (ELISA) (ELITEST, Hyphen, Neuville-sur-Olse, France), and the clinical outcome (asymptomatic and diseased). For gene expression microarray analysis, a total of 15 sheep were included; 11 of the animals were found naturally infected with VMV, six of them had lung lesions and the other five were considered seropositive for VMV but were asymptomatic. The remaining four animals were seronegative for VMV (Table 1). For the validation of the microarray analysis results, a total of 18 different animals were included (six seronegative, six seropositive asymptomatic and six with lung lesions) (Table 1). The samples were obtained from natural cases in the routine of the Veterinary Faculty (University of Zaragoza) in the framework of the national research project ref. AGL2010-22341-C04-01. The complete experimental procedure was approved and licensed by the Ethical Committee of the University of Zaragoza (ref: PI09/10). The care for and use of animals were performed according to the Spanish Policy for Animal Protection RD1201/05, which meets the European Union Directive 86/609 on the protection of animals used for experimental and other scientific purposes.

Animals were euthanized via an intravenous injection of a barbiturate overdose (Dolethal, Vetoquinol, Spain) and exsanguinated. A lung sample was aseptically taken from each animal and tissue sections were preserved in RNAlater solution (Ambion, Austin, TX, USA) at −80 °C until used.

### 2.2. RNA Isolation and Gene Expression Microarray Analysis

Total RNA was isolated from lung tissue using TRIzol Reagent (Invitrogen, Carlsbad, CA, USA). 60–70 mg tissue samples were homogenized in 1 mL of TRIzol Reagent using Precellys 24 homogenizer (Bertin Technologies, Montigny-le-Bretonneux, France) combined with 1.4 and 2.8 mm ceramic beads mix lysing tubes (Bertin Technologies). RNA isolation was performed following manufacturer instructions, and RNA was suspended in RNase free water and stored at −80 °C. RNA quantity and purity was assessed with a NanoDrop 1000 Spectrophotometer (Thermo Scientific Inc, Bremen, Germany). RNA integrity was assessed on an Agilent 2100 Bioanalyzer with Agilent RNA 6000 Nano chips (Agilent Technologies, Santa Clara, CA, USA), which estimates the 28S/18S (ribosomic RNAs) ratio and the RNA integrity number (RIN value).

RNA samples were labeled and hybridized following standard Agilent Protocol “One-Color Microarray-Based Gene Expression analysis (Low Input Quick Amp Labeling)” v 6.5. Briefly, 50 ng of total RNA were retrotranscribed and labeled using Low input Quick Amp Labeling kit one color (Agilent Technologies) following manufacturer instructions. First, total RNA was retrotranscribed with AffinityScript Reverse Transcriptase (Agilent Technologies), using Oligo dT primers coupled to a T7 promoter. Double stranded cDNA synthesized by AffinityScript RT was in vitro transcribed by T7 RNA pol in the presence of Cy3-CTP to generate amplified and labeled cRNA. Labeled samples were purified with silica-based RNeasy spin columns (Qiagen, Hilden, Germany). After labeling and purification, cRNA was quantified with NanoDrop 1000 spectrophotometer in order to determine the yield and specific activity of each reaction. Samples were analyzed using Agilent Sheep Gene Expression microarrays, and 8x15K (Design ID 019921) (Agilent Technologies), with 15,208 sheep features represented. 600 ng of labeled cRNA samples were hybridized at 65 °C for 17 h in a SureHyb hybridization chamber (Agilent Technologies). The arrays were scanned on a G2565CA DNA microarray scanner with ozone-barrier slide covers (Agilent Technologies). Scanned images were processed using Agilent Feature Extraction Software (v 10.7.3.1). Default parameters for one-color gene expression microarrays were used for image analysis, data extraction, background correction and the flagging of non-uniform features, population outliers in replicated features and with no significant intensities in Cy3 channel. Microarray data have been deposited in the GEO database with the accession number ESE107322. The RNA isolation and microarray analysis, including data preprocessing and statistical analysis, were performed at the Gene Expression Unit of the Genomics Facility, in the General Research Services (SGIker) of the University of the Basque Country (UPV/EHU).

### 2.3. Microarray Data Analysis

Raw data from Feature Extraction software were subsequently processed on GeneSpring GX 12.6 (Agilent Technologies). Data were normalized via quantile normalization for the elimination or correction of the systematic error due to technical variations and centered by mean to make gene expression from all microarrays comparable. After the normalization and mean centering of the data, non-uniform features, population outliers or features with intensities not significantly above background signal in 100% of samples in one out of any three conditions (Seronegative, Asymptomatic or Lesions) were filtered out.

For the identification of differentially expressed genes with statistical significance between groups, the LIMMA package was used [18]. The LIMMA statistical analysis was performed using MultiExperiment Viewer (MeV) v 4.7.1 (http://mev.tm4.org/) application. LIMMA applies the Benjamini–Hochberg method (FDR) for multiple test correction and obtains the adjusted *p* value. A multi-class comparison was conducted wherein all of the types of comparisons were performed: Asymptomatic vs. Seronegative, Lesions vs. Seronegative and Lesions vs. Asymptomatic.

### 2.4. Gene Ontology and Pathway Enrichment Analysis

In order to understand the biological significance of the results, the significant differentially expressed genes for each comparison (Lesions vs. Seronegative, Lesions vs. Asymptomatic) were analyzed for Gene ontology (GO) and pathway enrichment. First, the significant genes were annotated to the GO database for biological process (BP), cellular component (CC) and molecular function (MF) using the Blast2GO program [19]. Biological Process GO enrichment analysis was carried out on up-regulated and down-regulated significant genes from both comparisons using the FatiGO tool [20] implemented into the Babelomics web platform [21]. Significant enriched GO terms were clustered on different related functional groups using Database for Annotation, Visualization and Integrated Discovery (DAVID) (https://david.ncifcrf.gov/) [22]. KEGG pathway enrichment analysis was also conducted using DAVID on up-regulated and down-regulated significant gene lists.

### 2.5. Validation of Microarray Data by Reverse Transcription Quantitative PCR (RT-qPCR)

To validate the changes in gene transcription identified by the microarray experiment, the relative mRNA expression levels of 30 genes (selected based on significant changes seen in the microarray analysis and results of the GO enrichment analysis) were verified by reverse transcription quantitative PCR (RT-qPCR). Total RNA was isolated from a total of 18 Rasa sheep (Table 1) according to the protocol we used to prepare RNA for microarray analysis. RNA quantity and purity was assessed with NanoDrop 1000 Spectrophotometer (Thermo Scientific Inc.). RNA integrity and concentration was assessed with the 2100 Bioanalyzer (Agilent Technologies).

Specific primer pair sets for the 30 selected genes were designed using PrimerQuest and OligoAnalyzer tools of Integrated DNA Technologies (IDT) and in silico verified for non-specific annealing with Blast. Primer sequences for reference genes (ATPase, HPRT, GAPDH and YWHAZ) were obtained from Larruskain et al. [8], and primer sequences for TNF and C3 were obtained from Abendaño et al. [23] and Hillreiner et al. [24], respectively. Table S1 shows the list of the amplified ovine genes and the corresponding primer sequences. Primer pair specificity and primer dimer formation were verified by RT-qPCR using one of the samples. 600 ng of RNA were treated with DNAse I (Invitrogen) following the manufacturer’s instructions, and cDNA synthesis was carried out using the High Capacity cDNA Reverse Transcription Kit (Applied Biosystem, Foster City, CA, USA) following the manufacturer’s instructions with a mix of random primers and oligo (dT). qPCR reactions were accomplished using PowerUp SYBR Green Master Mix (Applied Biosystem) in a 10 µL final volume reaction according to the manufacturer’s instructions on a QuantStudio 3 detection system (Applied Biosystem) under the following conditions: 1 cycle of 50 °C for 2 min, 1 cycle of 95 °C for 2 min, 40 cycles of denaturation at 95 °C for 15 s and annealing at 60 °C for 60 s. The specificity of the primer pairs was verified via melting curve analysis following the last amplification cycle. Appropriate controls (no-template and minus-reverse transcriptase controls, samples in which no reverse transcriptase was added) were included. Primer concentrations that did not produce non-specific fragments or primer dimers and generated the lowest Ct value were selected for the final analysis. Primers designed for the CAV1, CD27, CXCL1 and SMAD7 genes were unsuccessful and were thus excluded from further analysis.

The expression of the “successful” 26 selected genes was measured using RT-qPCR in the 18 Rasa sheep (Table 1) RNA samples using Fludigm’s BioMark HD Nanofluidic qPCR System technology combined with GE 48.48 Dynamic Arrays IFC. The expression analysis (conducted with the Fluidigm Biomark HD Nanofluidic qPCR system) was performed at the Gene Expression Unit of the Genomics Facility, in the General Research Services (SGIker) of the UPV/EHU.

The cDNA was generated from 1 µg of total RNA using an AffinityScript Multiple Temperature cDNA Synthesis kit (Agilent Technologies) in a total volume of 20 µL, with a mix of random primers and oligo (dT). cDNA was used for specific target amplification (STA) using the Qiagen Multiplex PCR Master Mix and a mix of 50 nM primers. The PCR amplification conditions were 15 min at 95 °C followed by 14 cycles of 95 °C for 15 s and 60 °C for 4 min, followed by treatment with Exonuclease I (Exo I) (Thermo Scientific) to remove unincorporated primers (digestion, 37 °C for 30 min; enzyme inactivation, 80 °C for 15 min). Five-fold dilutions of STA reactions were loaded onto 48.48 Dynamic Array IFC according to Fluidigm’s Fast Gene Expression Analysis using EvaGreen protocol. SsoFastTM EvaGreen Supermix with Low ROX (Bio-Rad Laboratories) was used for amplification. The cycling program consisted of 1 min at 95 °C followed by 30 cycles of 95 °C for 5 s and 60 °C for 20 s, followed by a melting curve.

Five serial dilutions of a 1:10 factor of a control sample cDNA were run for the standard curve, used for evaluation of PCR amplification. Each sheep sample was run by duplicate and standard curve samples by triplicate. The software for the real-time PCR analysis and obtaining the Ct values was Fluidigm Real-Time PCR Analysis Software v 3.1.3.

### 2.6. RT-qPCR Data Analysis

PCR efficiency calculation and correction, reference gene stability analysis and normalization with selected reference genes were conducted with GenEx software v 5.4 of MultiD. PCR amplification efficiency was calculated from the slopes of the standard curves, according to the formula E = (10 ^(−1/slope)^) − 1, where “E” stands for the efficiency, and the slope is the gradient of the best fit line of the standard curve. Most of the genes had high efficiencies (90–106%), with a mean value of 97%. The stability of the candidate reference genes was analyzed using both NormFinder [25] and GeNorm [26] algorithms integrated in GenEx. The two most stable genes were HPRT and GAPDH, so normalization was performed using these two reference genes.

To determine the changes in gene expression (n-fold) or relative quantification (RQ), the following formula was used: RQ = 2^−Δ(ΔCT)^, where Δ*C_T_* is *C_T_* (target gene) − *C_T_* (reference gene) and Δ(Δ*C_T_*) is Δ*C_T_* (experimental) − Δ*C_T_* (control). Based on the microarray results, two comparisons were conducted: Lesions vs. Seronegative and Lesions vs. Asymptomatic.

Results were expressed as relative quantification, and fold changes were standardized by log2 transformation. Normal distribution was checked using the Kolmogorov–Smirnov test in the SPSS statistical package v 24 (IBM Statistics v 24). Changes in gene expression between different groups (Lesions vs. Seronegative and Lesions vs. Asymptomatic) were compared with the Tukey HSD or Games–Howell post-hoc test (ANOVA) or with a non-parametric Kruskal–Wallis test of the SPSS package. Statistical significance of the comparison between results obtained with microarray and RT-qPCR was calculated by using a Wilcoxon signed rank test. Correlation between the microarray and the RT-qPCR results was performed using Spearman’s Rho non-parametric test or Pearson’s correlation, depending on the normality of the data. In all analyses, differences were considered significant when *p* values were <0.05.

## 3. Results

### 3.1. Gene Expression Microarray Analysis

There are 15,008 non-control probes or biological features in the sheep array design employed. However, after filtering out for the non-uniform features, population outliers and features with intensities not significantly above background signal in 100% of samples in one out of any three conditions (Seronegative, Asymptomatic, Lesions), 12,823 features were retained for further statistical analysis. The selected genes were used to generate a PCA to visualize the 15 samples (Figure S1). In the PCA, the samples were grouped according to the animal’s clinical status (Lesions, Asymptomatic, and Seronegative).

**Figure 1:**
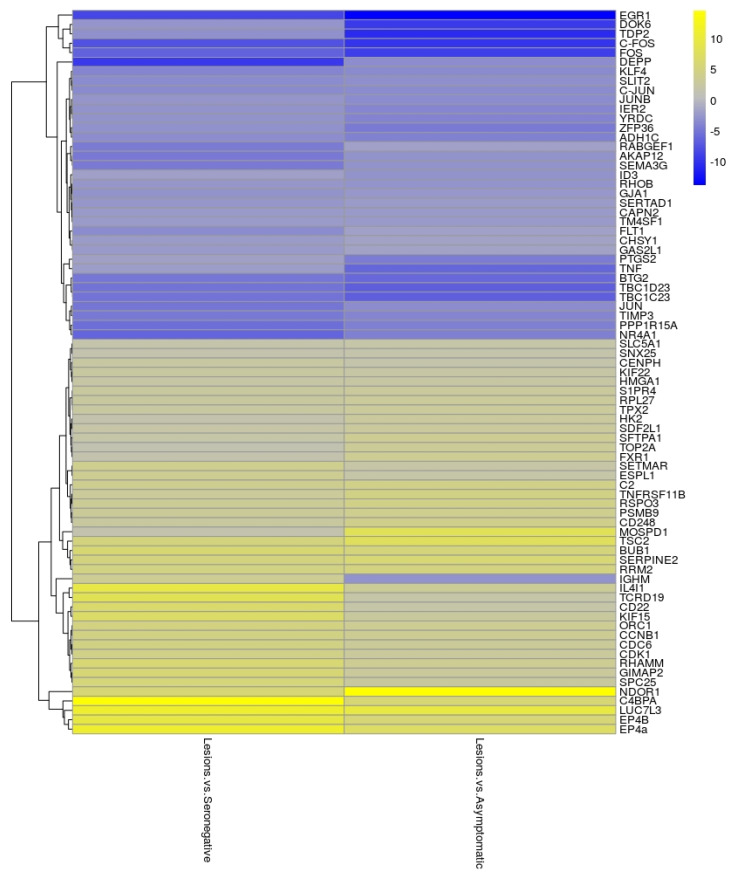
Heat-map of the most significant up- and down-regulated genes in the Lesions group relative to the Seronegative or Asymptomatic group. A blue color indicates genes’ down-regulation while a yellow color indicates genes’ up-regulation. The color intensity from blue to yellow indicates the gene expression levels.

Microarray data analysis revealed that there were no significant changes in gene expression between the Asymptomatic and Seronegative groups (adjusted *p*-value from LIMMA test > 0.05). However, a large number of genes (*n* = 470; adjusted *p* values < 0.05) were differentially regulated in the Lesions group as compared to those of the Seronegative group. Among these, 266 were up- and 204 were down-regulated. 593 genes were also found to be differentially expressed (adjusted *p* values < 0.05) in the Lesions group, compared to the Asymptomatic group, 260 being up- and 333 down-regulated. The lists of significant differentially expressed genes in both comparisons (Lesions vs. Seronegative and Lesions vs. Asymptomatic) were compared with Venn diagrams (Figure S2). The top 25 significant up- and down-regulated genes in Lesions group relative to the Seronegative group or the Asymptomatic group are shown as a heatmap (Figure 1) and a companion table (Table S2). Within the most up- or down-regulated genes, factors clearly related to cell cycle (BUB1, CDC6, CCNB1, CDK1, ESPL1, ORC1), apoptosis (NR4A1, RHOB, SLIT2, CAPN2), complement system (C2, C4BPA), cytokine–cytokine receptor interaction (TNFRSF11B, FLT1) and TNF signaling pathway (FOS, JUN, JUNB, PTGS2 and TNF) were identified.

### 3.2. Quantitative PCR Validation

**Figure 2:**
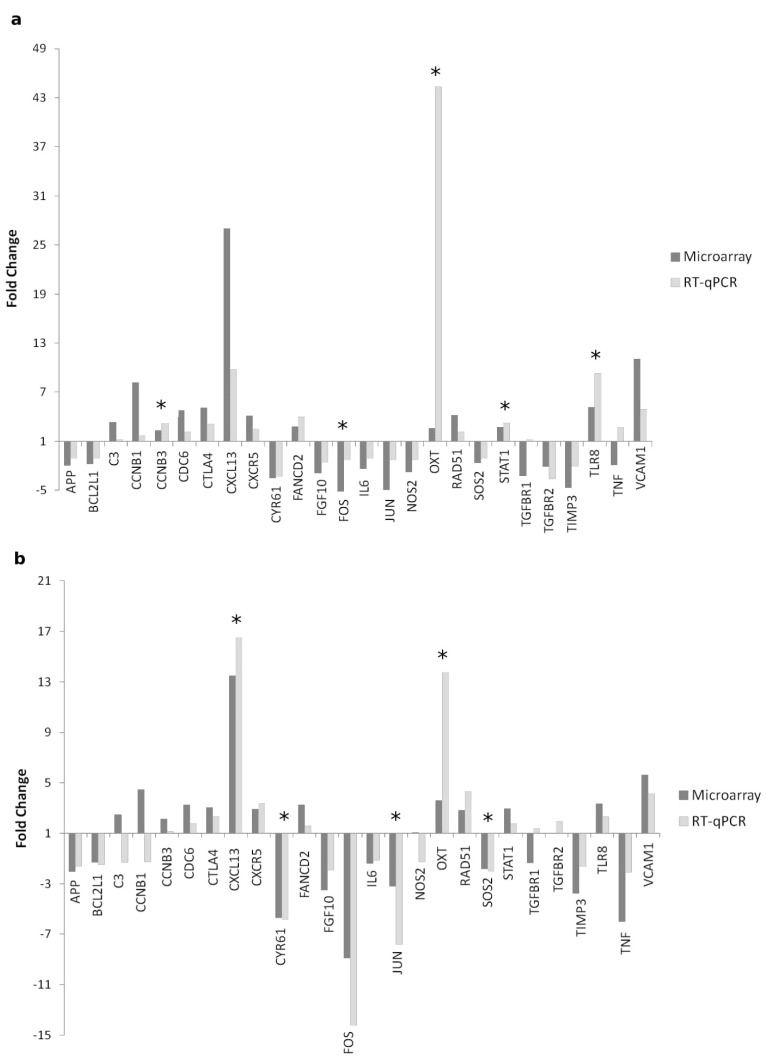
Expression of the selected 26 genes in a. Lesions group relative to Seronegative group b. Lesions group relative to Asymptomatic group measured by microarray and RT-qPCR. Bars represent the difference in Fold Change between groups of animals compared. Fifteen animals were included in the expression microarray and 18 animals in the RT-qPCR analysis. Statistically significant differences in the expression measured by RT-qPCR of the indicated genes are showed with an asterisk (*p* < 0.05).

To validate changes in gene transcription identified by the microarray experiment, the relative mRNA expression levels of the selected 26 genes were verified via RT-qPCR using the Fluidigm Biomark HD Nanofluidic qPCR system. Fold changes in gene expression between Lesions vs. Seronegative and Lesions vs. Asymptomatic groups calculated by RT-qPCR are shown in Figure 2. RT-qPCR expression analysis revealed nine genes differentially expressed (CCNB3, CXCL13, CYR61, FOS, JUN, OXT, SOS2, STAT1 and TLR8) in a statistically significant way across multiple groups. Variations in the expression levels of five genes were statistically significant in Lesions group relative to Seronegative group. Four of these genes were up-regulated, CCNB3 (*p* = 0.043), OXT (*p* = 0.000), STAT1 (*p* = 0.007) and TLR8 (*p* = 0.000), whereas FOS was down-regulated (*p* = 0.041). In addition, if animals with Lesions were compared to Asymptomatic animals, CXCL13 and OXT were significantly up-regulated (*p* = 0.027, *p* = 0.001). In contrast, a significantly down-regulated expression was observed for CYR61 (*p* = 0.015), JUN (*p* = 0.041) and SOS2 (*p* = 0.009).

Although the fold change values for the expression of some genes measured via microarray or RT-qPCR were different (C3, CCNB1, NOS2, TGFBR1 and TNF), in terms of fold change direction, the gene expression patterns of most of the genes (APP, BCL2L1, CCNB3, CDC6, CTLA4, CXCL13, CXCR5, CYR61, FANCD2, FGF10, FOS, IL6, JUN, OXT, RAD51, SOS2, STAT1, TGFBR2, TIMP3, TLR8 and VCAM1) were reproducible via RT-qPCR analysis, and there were no significant differences in fold change data obtained with microarray and the Fluidigm Biomark HD Nanofluidic qPCR system (*p* > 0.05). Data from microarray and RT-qPCR showed a high degree of concordance (Figure 2), with a correlation coefficient of 0.724 (*p* = 0.01) in Lesions vs. Seronegative comparison and 0.801 (*p* = 0.01) in Lesions vs. Asymptomatic comparison.

### 3.3. Gene Ontology and Pathway Analysis

**Figure 3:**
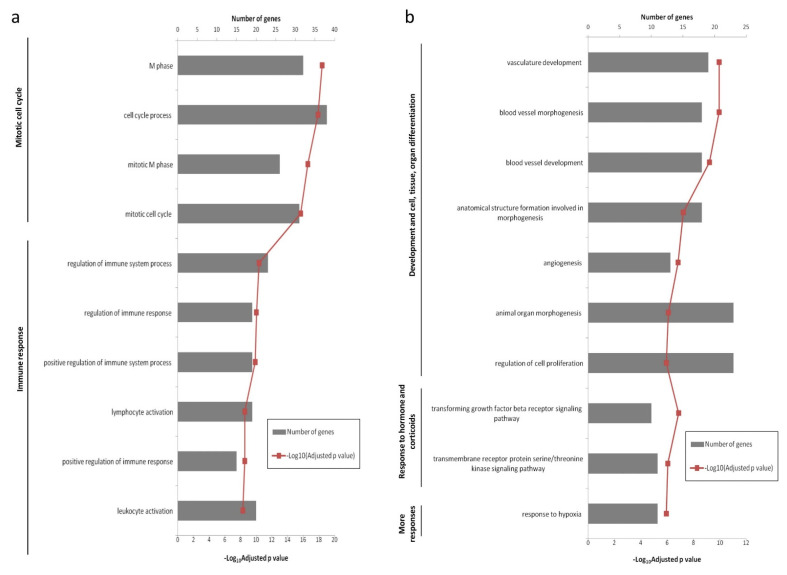
The Top 10 significant enriched GO terms in the Lesions group relative to the Seronegative group. (**a**) Up-regulated genes (**b**) Down-regulated genes.

**Figure 4:**
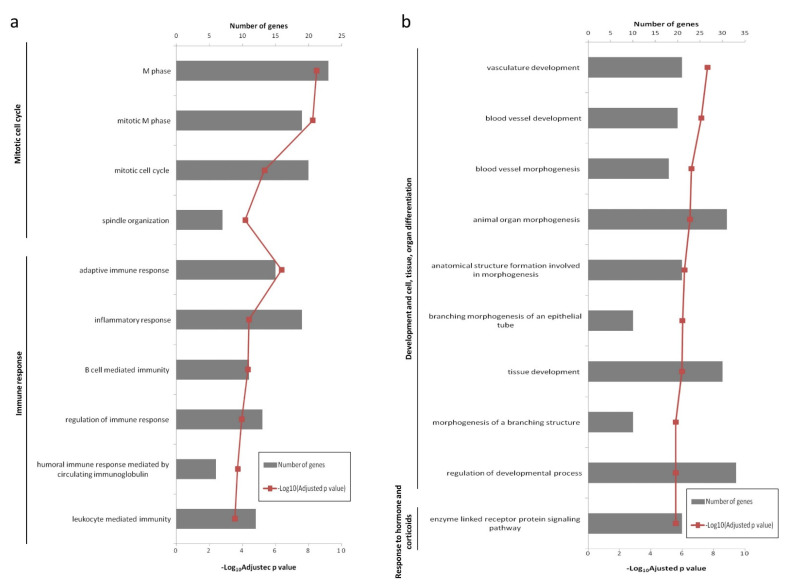
The Top 10 significant enriched GO terms in the Lesions group relative to the Asymptomatic group. (**a**) Up-regulated genes (**b**) Down-regulated genes.

**Figure 5:**
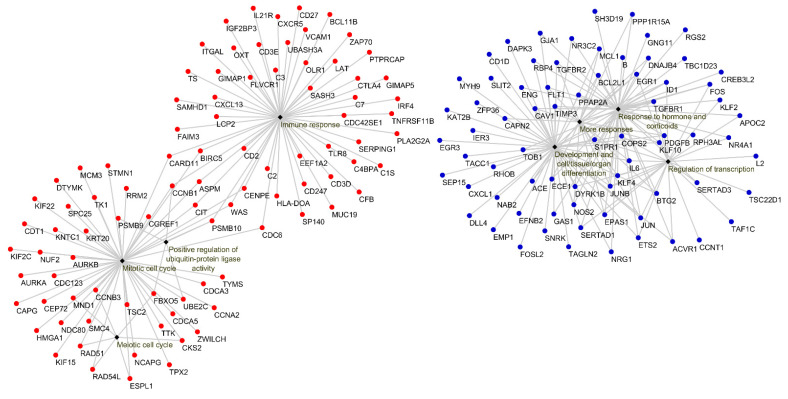
Enriched functional GO terms groups obtained with FatiGO (Babelomics 4.3) in the significant up-regulated (red) and down-regulated (blue) genes when comparing the Lesions group with the Seronegative group. *p* value is the highest adjusted *p* value of its groups GO terms: Mitotic cell cycle (*p* = 3.91 × 10^−19^), Meiotic cell cycle (*p* = 1.77 × 10^−3^), Immune response (*p* = 4.14 × 10^−11^), Positive regulation of ubiquitin-protein ligase activity (*p* = 9.14 × 10^−3^), Development and cell/tissue/organ differentiation (*p* = 1.11 × 10^−10^), Regulation of transcription (*p* = 7.97 × 10^−5^), Response to hormone and corticoids (*p* = 1.35 × 10^−7^), More responses (*p* = 1.16 × 10^−6^).

**Figure 6:**
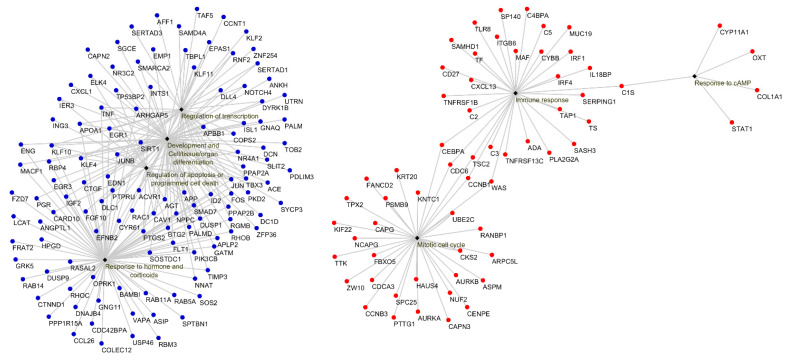
Enriched functional GO terms groups obtained with FatiGO (Babelomics 4.3) in the significant up-regulated (red) and down-regulated (blue) genes when comparing the Lesions group with the Asymptomatic group. *p* value is the highest adjusted *p* value of its groups GO terms: Mitotic cell cycle (*p* = 3.30 × 10^−9^), Immune response (*p* = 4.35 × 10^−7^), Response to cAMP (*p* = 6.02 × 10^−3^), Development and cell/tissue/organ differentiation (*p* = 2.33 × 10^−8^), Regulation of transcription (*p* = 6.51 × 10^−6^), Response to hormone and corticoids (*p* = 2.54 × 10^−6^), Regulation of apoptosis or programmed cell death (*p* = 1.34 × 10^−3^).

Biological process GO terms and pathway enrichment analyses were performed using the lists of differentially expressed genes identified by the microarray analysis in the Lesions group relative to the Seronegative group or the Asymptomatic group. GO enrichment analysis revealed that 66 terms were enriched in the up-regulated genes in Lesions group vs. Seronegative group. Additionally, 67 functions were enriched in the down-regulated mRNAs for the same comparison. In the Lesions vs. Asymptomatic comparison, 21 GO terms were enriched in the up-regulated genes, whereas 67 functions were enriched in down-regulated genes. The top 10 significant GO terms in Lesions group relative to Seronegative group and Asymptomatic group are shown in Figure 3 and Figure 4, respectively. Enriched GO terms were clustered on related functional groups with DAVID. Enriched GO terms in the up-regulated gene lists were related to the regulation of mitotic cell cycle, meiotic cell cycle, immune response (inflammatory response, innate immune response, B-cell mediated immune response, T-cell mediated immune response), the positive regulation of ubiquitin-protein ligase activity and response to cAMP. In contrast, enriched GO terms in the down-regulated genes were related to the regulation of development and cell/tissue/organ differentiation, regulation of transcription, response to hormone and corticoids, regulation of apoptosis or programmed cell death and more responses (response to hypoxia, response to mechanical, protein and cytokine stimulus and response to organic nitrogen). Genes involved in each functional cluster of the enriched GO terms in the comparisons Lesions group vs. Seronegative group or Asymptomatic group are shown in Figure 5 and Figure 6, respectively.

In addition, KEGG pathway enrichment analysis revealed that differentially expressed genes are involved in pathways of complement and coagulation cascades, T cell receptor signaling pathway, cell cycle, TNF signaling pathway, osteoclast differentiation, PI3K-Akt signaling pathway, signaling pathways regulating pluripotency of stem cells, MAPK signaling pathway and TGFB signaling pathway (Table S3).

## 4. Discussion

As far as we know, this is the first study on mRNA profiles in sheep infected with VMV and with disease lesions. To identify the differentially expressed genes during the infection with VMV, we used 15 animals belonging to three groups: seronegative, seropositive but asymptomatic and diseased (with lesions) animals; accordingly, 3 comparisons were made: asymptomatic vs. seronegative, diseased vs. seronegative and diseased vs. asymptomatic.

Cytokines seem to have an important role in SRLV induced pathogenesis. In this study 3 cytokines were found to be differentially regulated: IL6, TNF and IFN-*γ*. IL6 was down-regulated in infected and asymptomatic animals compared with seronegative animals, and significantly downregulated in the Lesions vs. Seronegative comparison, as in other studies in goat macrophages infected with CAEV [27] and in the whole blood of infected goats [28]. As previously reported [8,9], the pro-inflammatory cytokine TNF has been found down-regulated in the lung of VM clinically affected animals (Lesion vs. Seronegative comparison). However, IFN-*γ* was up-regulated and presented a fold change of 5.05 in the Lesions vs. Asymptomatic comparison. This result does not match with previous studies, as it has been reported that SRLVs seldom induce type I IFN production directly from infected cells [29]. Our information on interferons is limited to IFN-*γ* so further studies are needed on this important mediator of immunity against virus infections.

Interestingly a change in differential expression was detected in the IGHM gene, related with antibody-mediated immune response. In this work, it was up-regulated in the Lesions group relative to the Seronegative group and significantly down-regulated in animals with lesions comparing with asymptomatic animals. This gene encodes the constant region of the mu heavy chain, which defines the IgM antibody subtype. IgM antibodies play an important role in primary defense mechanisms and sheep have been found to respond to acute viral infections by an initial formation of the IgM class antibodies followed in a couple of weeks by the formation of IgG type [30]. This would explain the up-regulation detected in the first steps of the infection.

In relation to the innate immune response, TLR8 was significantly up-regulated both in asymptomatic animals and in animals with VM lung lesions (*p* < 0.05). It is known that TLR8 plays a fundamental role in pathogen recognition and activation of innate immunity, recognizing pathogen-associated molecular patterns (PAMPs) that are expressed on infectious agents and mediating the production of cytokines. This finding is in agreement with previous reports of our group, as well as others that also found TLR8 to be up-regulated in the lungs of animals with lesions compared to control animals [13,31].

Furthermore, three other genes were down-regulated in the Lesions group compared with asymptomatic animals, PTGS2 and IER3 (both involved in regulation of apoptosis and cell and tissue differentiation), and UTRN, a gene associated with cell, tissue and organ development. UTRN has been found to be associated with susceptibility and serologic status of Ovine Lentivirus (OvLV) [7] and Thompson et al. [10] also found that Ovine Progressive Pneumonia Virus (OPPV) infection causes decreasing expression of PTGS2 and IER3 in *Capra hircus in vitro*. Except for Interferons, our results confirm previous findings, some of them in goats, in relation to differential regulation of genes in response to viral infection.

At last, we also found some genes that were known to be associated with interference against the human retrovirus HIV, such as CTLA4 [32], TLR8 [33], SAMHD1 [34]; BCL2L1 [35]; ITGAL and JUN [36]; FOSL2 [37] and STAT1 [38]. The gene expression changes of all these genes were in accordance with the changes observed in our study. In particular, CTLA4, TLR8, SAMHD1, ITGAL and STAT1 were significantly up-regulated and, in contrast, a significant down-regulated expression was observed for BCL2L1, FOSL2 and JUN when we compared animals with lesions to seronegative and asymptomatic animals. Interestingly, SAMHD1 was one of the restriction factors of the innate immune response identified in this study, which warrants future research on this protein related to SRLV infection and its potential to restrict viral infection.

Microarray data analysis as a whole revealed that there were not significant differentially expressed genes with adjusted *p*-values < 0.05 in Asymptomatic vs. Seronegative comparison. However, 470 genes were significantly differentially regulated in the Lesions group compared to the Seronegative group. Of these genes, 266 were up-regulated and 204 were down-regulated. 593 genes were also found to be significantly differentially expressed in the Lesions group compared to the Asymptomatic group; 260 being up-regulated and 333 being down-regulated. The lack of differentially expressed genes with corrected *p*-values in the Seronegative vs. Asymptomatic comparison could be related with the analysis of naturally infected samples instead of animals infected experimentally, as is usually done in this kind of works. Negative animals were classified according to ELISA test which does not reach 90% of sensitivity and therefore false negative could be expected in the serologic test [39]. At last, natural infection could have occurred at different times, biasing data and interpretation.

To confirm changes in gene transcription identified by the microarray experiment, the relative mRNA expression levels of 26 genes were verified by Fluidigm Biomark HD Nanofluidic qPCR system in different samples, with animals classified in three groups. The gene expression patterns of most of the selected genes (90% of the comparisons) were reproducible via RT-qPCR analysis, validating the results obtained in the microarray analysis. Surprisingly, the OXT gene appeared much more up-regulated in the qPCR analysis. Recently, the hypothalamo–neurohypophysial system emerged as an important component of the neuroendocrine–immune network, wherein the oxytocin-secreting system (OSS) plays an essential role. The OSS can promote the development of thymus and bone marrow, perform immune surveillance, strengthen immune defense and maintain immune homeostasis [40]. Oxytocin can strengthen the physical and chemical barriers through suppressing pro-inflammatory cytokines [41], and many of them were down-regulated in the array results. Thus, it is quite possible that OSS could affect the response against VMV.

Similar results were obtained in the biological process GO terms enriched analysis of the up-regulated and down-regulated genes for the two comparisons with significant differences, Lesions group vs. Seronegative group and vs. Asymptomatic group. This analysis reveals that VMV induces significant up-regulation of genes implicated in the mitotic cell cycle and immune response in both comparisons. In addition, meiotic cell cycle and positive regulation of ubiquitin-protein ligase activity GO terms were enriched in up-regulated genes when we compared Lesions group vs. Seronegative group. Recently, the mechanism by which Vif proteins of bovine immunodeficiency virus (BIV) facilitate viral infection has been described [42]. It differs from the mechanism of primate viruses because it requires different ubiquitin ligase scaffolding proteins. Extended information should be needed to disentangle if this mechanism is also used by VMV in sheep.

KEGG pathway analysis found several enriched pathways. In Lesions vs. Asymptomatic comparison, six pathways were found to be significantly enriched. Two were enriched in up-regulated genes (complement and coagulation cascades and cell cycle) and four were enriched in the down-regulated genes: TNF signaling pathway, signaling pathways regulating pluripotency of stem cells, MAPK signaling pathway and TGFB signaling pathway. Moreover, in Lesions vs. Seronegative comparison, T cell receptor signaling pathway was enriched in up-regulated genes and PI3K-Akt signaling pathway in the down-regulated genes.

Some differentially regulated genes involved in these pathways deserve special attention. In Lesions vs. Asymptomatic comparison, not only TNF but seven other genes implicated in TNF signaling pathway were down-regulated: CXCL1, FOS, PTGS2, PIK3CB, JUN, EDN1, JUNB. In contrast, C3, C5, C2, C1S, C4BPA (involved in the complement pathway) and SERPING1 (a regulator of complement activation) were significantly up-regulated. The same differential regulation was also detected in the Lesions vs. Seronegative group comparison, and SERPING1 overexpression has been recently described in monocytes from HIV patients [43]. The complement system functions as an immune surveillance system that rapidly responds to infection, being a critical determinant of the infection outcome by a variety of different viruses [44], and this could also be the case for SRLVs. In addition, the complement system is intricately linked with other pathways that play important roles in viral pathogenesis, such as the Toll-like receptor signaling network [45]. The up-regulation of TLR8 could support this mechanism in the VM pathogenesis.

## 5. Conclusions

Our study using microarray technology to search for host cellular association factors during VMV infection (1) confirms some of the previously described differentially expressed genes (mainly some cytokines such as TNF and IFN-*γ*) and (2) provides relevant information about new genes and pathways involved in the infection and pathogenesis of the VMV. The OXT gene appeared significantly up-regulated, so the oxytocin-secreting system could play an essential role. Moreover, C3, C5, C2, C1S, C4BPA (genes involved in the complement pathway) and SERPING1 (a regulator of complement activation) were significantly up-regulated, indicating an important role of the complement pathway in this infection.

The discovery of new genes associated with viral infection and pathogenesis can help to understand the mechanisms of the infection and the development of the disease provoked by SRLVs.

## Figures and Tables

**Table 1:** Samples used in Microarray and RT-qPCR study.

Microarray	
Status	Animals (15)
Pulmonary lesions	1P, 2P, 4P, 7P, 9P, 10P
Seropositive asymptomatic	8P, 11P, 12P, P17, P19
Seronegative	P13, P14, P15, P16
**RT-qPCR**	
**Status**	**Animals (18)**
Pulmonary lesions	P21, P22, P24, P25,P26,P27
Seropositive asymptomatic	1, 2, 3, 4, 5, 6
Seronegative	7, 10, 11, 12, 13, 14

## Data Availability

Microarray data have been deposited in the GEO database with accession number ESE107322.

## Supplementary Materials

The following are available online at https://www.mdpi.com/article/10.3390/ani11061785/s1, Table S1: List of the selected genes and the corresponding primer sequences for the validation of the microarray, Table S2: The Top 25 significant up- and down-regulated genes in Lesions group relative to Seronegative or Asymptomatic group, Table S3: Significant enriched KEGG pathways for differentially expressed genes in Lesions group relative to Seronegative or Asymptomatic group, Figure S1: PCA plot of microarray data from six animals with pulmonary lesions, five asymptomatic animals and four seronegative animals, Figure S2: Venn diagrams showing the number of genes identified as differentially expressed.
