# Supplementary figures and images for: Gene Expression Profiling Reveals New Pathways and Genes Associated with Visna/Maedi Viral Disease
Source: Animals (Basel). 2021 Jun 15;11(6):1785. doi: 10.3390/ani11061785 (PMC8232142; doi:10.3390/ani11061785)

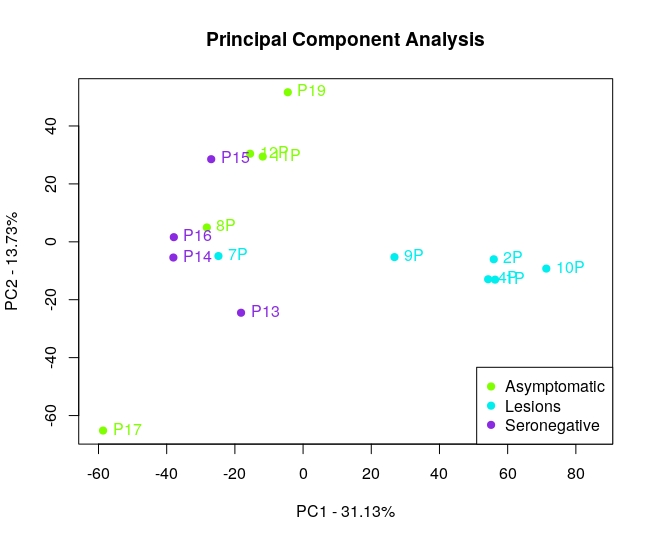

Supplement: Supplementary file 1 [file animals-11-01785-s001.zip › Figure S1.tif]

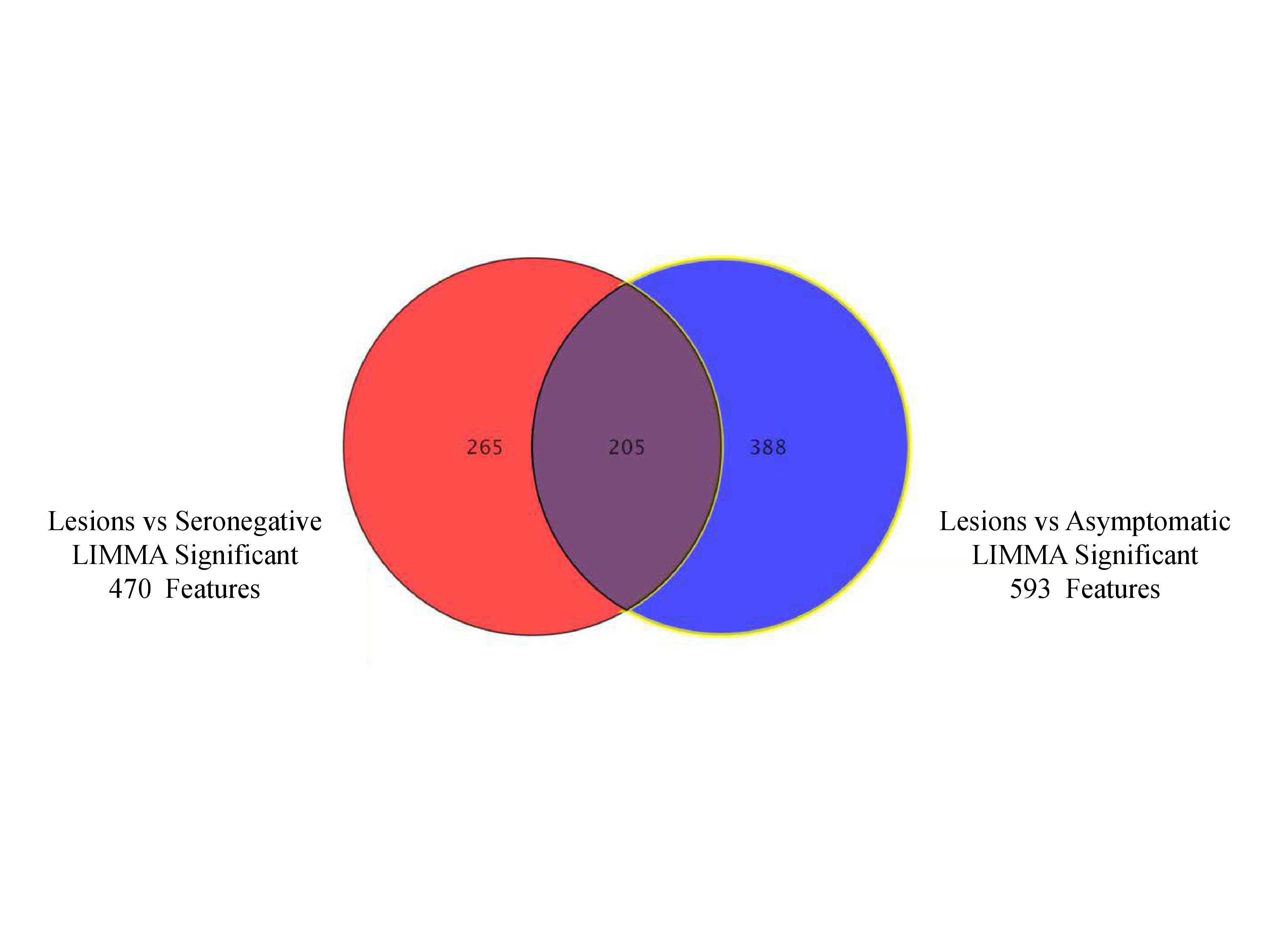

Supplement: Supplementary file 1 [file animals-11-01785-s001.zip › Figure S2.tif]
